# Transcriptomic and metabolomic analysis based on different aggressive pecking phenotype in duck

**DOI:** 10.1038/s41598-024-73726-9

**Published:** 2024-09-27

**Authors:** Baoguo Zhu, Jinjin Zhu, Ai Liu, Bingnong Yao, Fuyou Liao, Shenglin Yang

**Affiliations:** 1 Key Laboratory of Animal Genetics, Breeding and Reproduction in the Plateau Mountainous Region, Ministry of Education, Guizhou University, Guiyang, 550025 China; 2 Key Laboratory of Animal Genetics, Breeding and Reproduction of Guizhou province, Guizhou University, Guiyang, 550025 China; 3 College of Animal Science, Guizhou University, Guiyang, 550025 China

**Keywords:** Duck, Aggressive pecking, Transcriptomic, Metabolomic, Metabolomics, RNA sequencing, Animal behaviour

## Abstract

Aggressive pecking is an important welfare and production efficiency issue in poultry farming. The precise mechanisms underlying the occurrence of aggressive pecking remain poorly understood. In this study, we selected Sansui ducks that performed aggressive pecking and ducks that did not perform aggressive pecking from video recordings. Transcriptomic and metabolomic analyses of the whole brains of aggressive pecking ducks and normal ducks revealed 504 differentially expressed genes and 5 differentially altered metabolites (adenosine, guanidinopropionic acid, Met-Leu, Glu-Ile and 5,6,8-trihydroxy-2-methylbenzo[g]chromen-4-one). By jointly analysing the transcriptomics and metabolomics results, we discovered 8 candidate genes (*ADCYAP1*, *GAL*, *EDN2*, *EDN1*, *MC5R*, *S1PR4*, *LOC113843450*, and *IAPP*) and one candidate metabolite (adenosine) that regulates aggressive pecking behaviour in ducks. The candidate genes and metabolites may be involved in regulating aggressive pecking behaviour by inducing neurodegeneration and disrupting neural excitatory-inhibitory homeostasis, which in turn affects central nervous system function in aggressive pecking and normal ducks. Our findings provide a new reference for revealing the underlying mechanism of aggressive pecking behaviour in ducks.

**Supplementary Information:**

The online version contains supplementary material available at 10.1038/s41598-024-73726-9.

## Introduction

Ducks constitute one of the major sources of animal protein production. For the purpose of increasing duck production and achieving profitability, managers need to improve the performance of ducks through their housing system^1^. However, housing systems are among the key factors affecting animal welfare. An elevated stocking density diminishes the available space for individual poultry, thereby reducing the living resources at their disposal. As a consequence, poultry are rendered more susceptible to distress, with the potential for a higher level of stress resulting in a greater likelihood of aggressive behaviour^2^. Aggression is defined as the behaviour of individual animals fighting each other under conditions conducive to individual survival. This competitive behaviour is influenced by multiple factors, including food, mates, social status, and others. The aggressive behaviours of poultry are primarily categorized as threatening, chasing, aggressive pecking (AP) and clawing^3^.

AP behaviour, which is usually directed at the head of the receiving bird in poultry, is an important part of their daily life, and is used to some extent to build and maintain social hierarchies in the group^4^. However, AP is also an important welfare and production efficiency issue in poultry farming^5^. If not stopped in time, AP can cause social stress, physical injury, and death, leading to serious economic loss^6^. A number of environmental factors have been identified as influencing AP, including light^7^, stocking density^8^, food^9^, feeding methods^10^, and group size^11^. To date, the underlying mechanisms responsible for the occurrence of AP in ducks remain poorly understood. Although some previous studies have shown that AP is associated with postsynaptic signalling through genome-wide association analyses^12^ and microarray analyses^13^, the transcriptomics and metabolome of AP in ducks remain unexplored. Transcriptomics is a powerful technology that provides insight into the complexity of gene expression in organisms. The metabolome is downstream of gene regulatory networks and protein action networks, and provides terminal information about biology. By integrating transcriptomics and metabolomics studies, the underlying mechanisms of AP can be better revealed. Therefore, exploring the mechanism of AP behaviour in ducks through transcriptome and metabolome profiling is useful for preventing and reducing the occurrence of excessive AP behaviour in intensive farming.

In this study, we observed the AP of Sansui ducks on video and selected Sansui ducks that performed AP and ducks that did not perform AP. We subsequently screened the differentially expressed genes (DEGs) and differentially altered metabolites (DAMs) in the whole brains of AP ducks and Normal ducks by transcriptomics and metabolomics. Finally, we identified 8 candidate genes and 1 candidate metabolite by combining the results of transcriptomics and metabolomics. These findings will contribute to our understanding of the mechanisms underlying AP behaviour in ducks.

## Results

### Transcriptome quality assessment

The quality control results of the whole-brain transcriptomes are listed in Table 1. Six ducks from the AP and Normal group were used to construct cDNA libraries. A total of more than 34 million valid reads were obtained from each library. The GC contents of all the samples ranged from 46.5 to 47%, with the percentage of the Q20 bases ranged from 98.43 to 98.72%, and the percentage of the Q30 bases ranged from 94.02 to 95.02%.

**Table 1:** Quality control results of transcriptome.

Sample	Valid reads	Valid bases	Valid ratio (%)	Q20 (%)	Q30 (%)	GC (%)
AP Group_1	39,659,902	5.95G	93.95	98.71	94.81	47
AP Group_2	45,393,432	6.81G	94.55	98.69	95.02	46.5
AP Group_3	39,183,016	5.88G	93.71	98.43	94.02	46.5
Normal Group_1	34,944,968	5.24G	95.57	98.66	94.94	46.5
Normal Group_2	35,405,148	5.31G	95.3	98.55	94.31	46.5
Normal Group_3	38,617,052	5.79G	94.7	98.72	94.74	46.5

### Transcriptome analyses

**Fig. 1:**
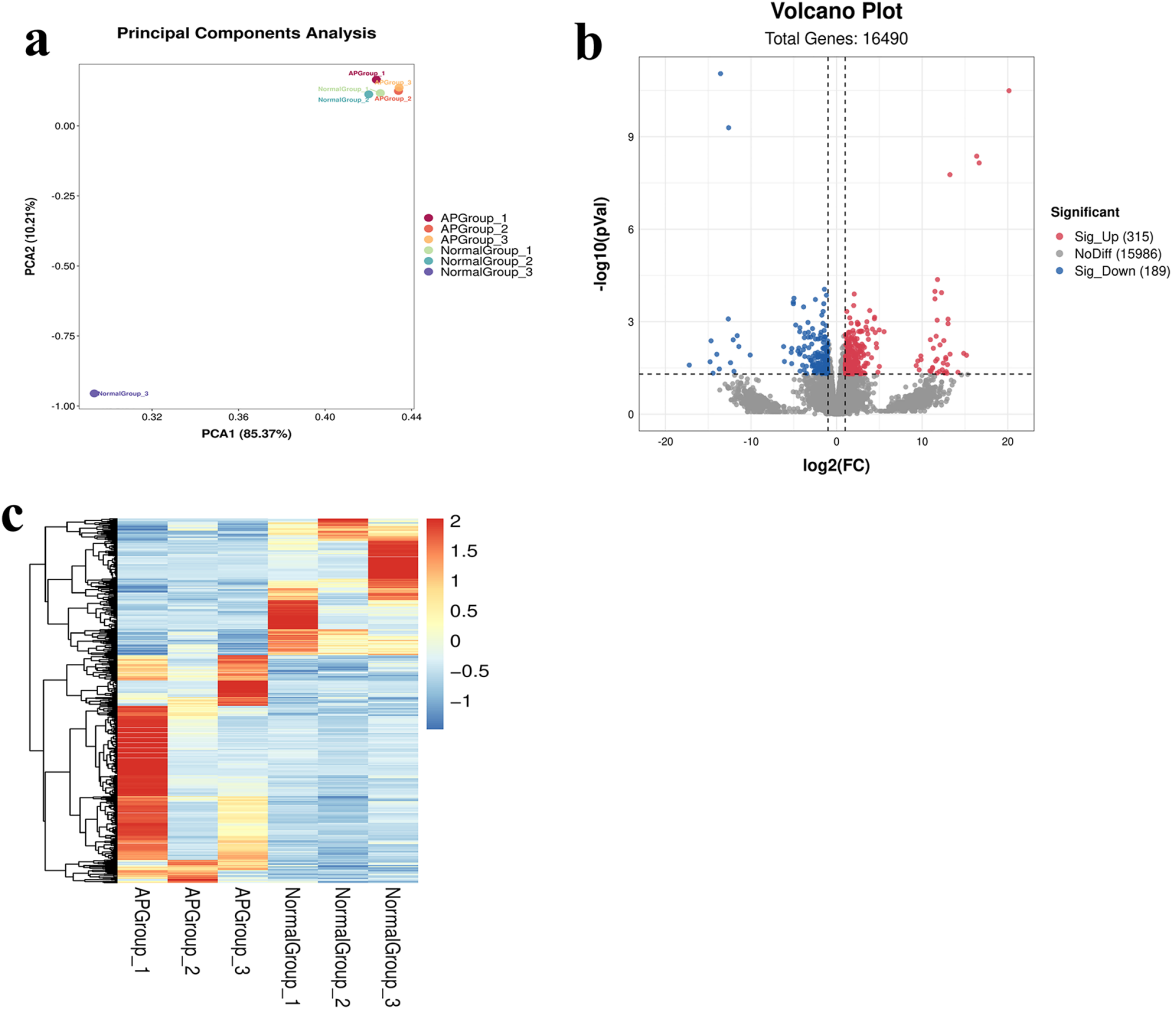
Transcriptome analysis results. (**a**) PCA plot. (**b**) Volcano plot of DEGs: the red plots represent significantly upregulated genes; the blue plots represent significantly downregulated genes. (**c**) Clustering heatmap of the transcriptome.

As shown in the principal component analyses (PCA) plot (Fig. 1a), the cumulative values of PCA1 and PCA2 were 95.58%, indicating the presence of DEGs in the AP and Normal groups. To explore DEGs in the whole brains of AP ducks and Normal ducks, gene expression levels were quantified by fragment per kilobase of transcript per million mapped reads (FPKM). A total of 504 DEGs (315 upregulated DEGs and 189 downregulated DEGs, Supplementary Table S1) were identified between the AP and Normal groups (Fig. 1b). These DEGs are potentially regulators of AP behaviour. In addition, a clustered heatmap was generated based on the expression levels of the DEGs (Fig. 1c).

### Gene enrichment analyses

**Fig. 2:**
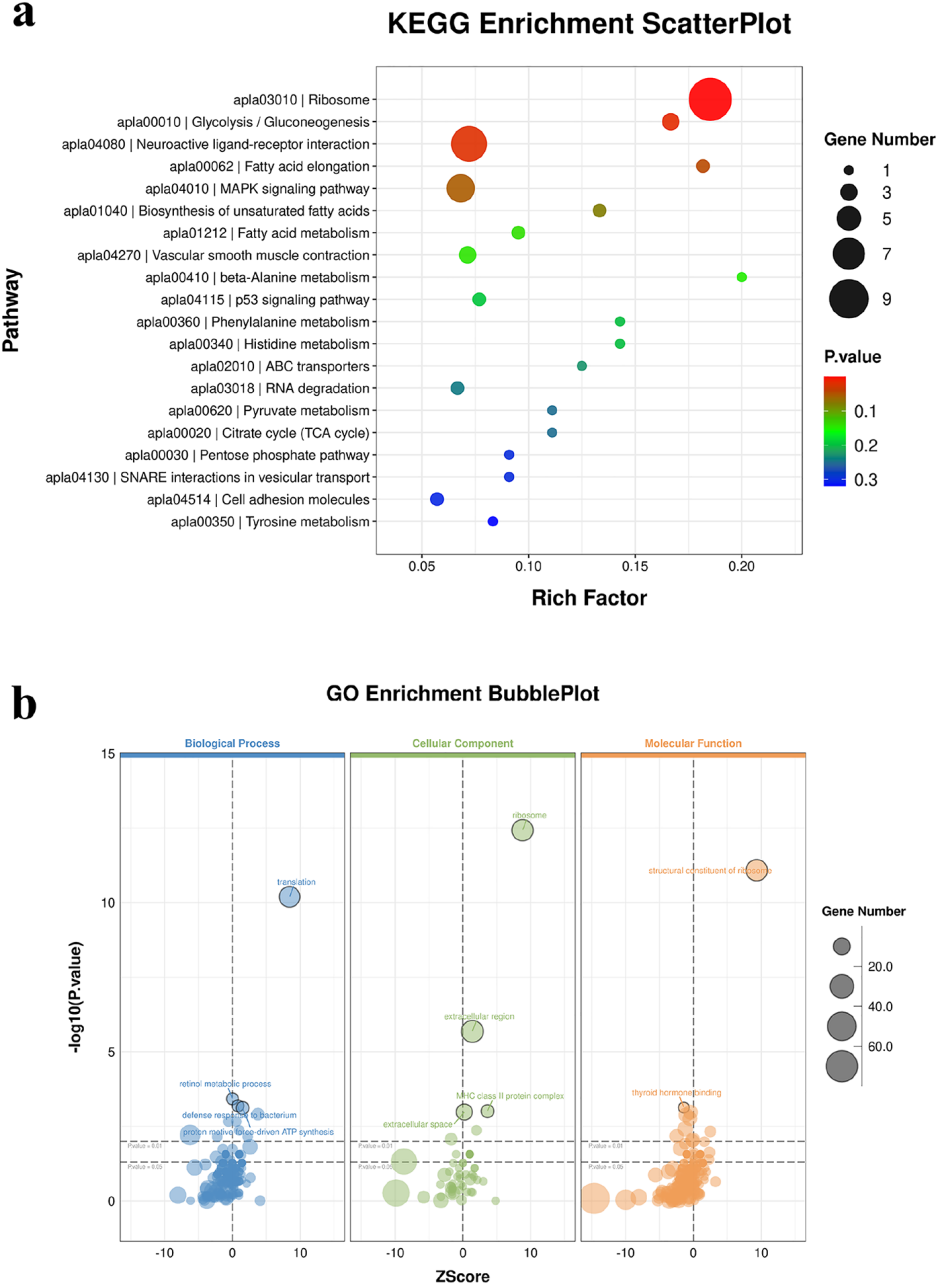
DEGs enrichment analysis plots. (**a**) KEGG enrichment scatter plot. (**b**) GO enrichment bubble plot.

To investigate the potential roles of these DEGs, Kyoto Encyclopedia of Genes and Genomes (KEGG) enrichment analysis were performed as well as Gene Ontology (GO) analysis. KEGG enrichment analyses (Fig. 2a) showed that the DEGs were significantly enriched in ribosomes, glycolysis/gluconeogenesis, neuroactive ligand-receptor interaction, and fatty acid elongation pathways. In addition, GO enrichment analysis (Fig. 2b) showed that the DEGs were significantly enriched in biological processes such as translation and retinol metabolic process. GO enrichment was also observed for cellular components and molecular functions, which included GO terms such as ribosomes, structural components of ribosomes, extracellular region and thyroid hormone binding.

### Metabolome analysis

**Fig. 3:**
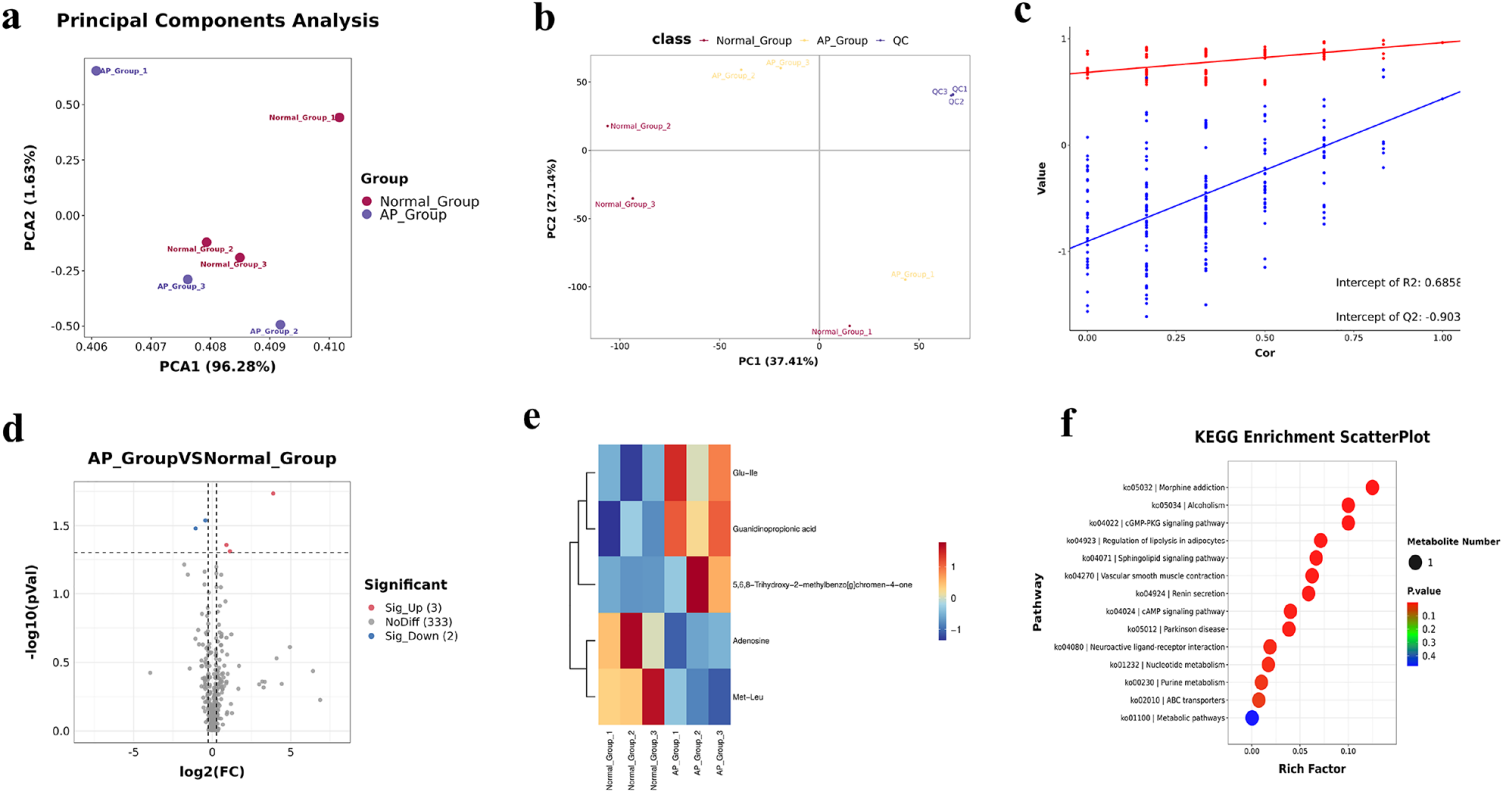
Metabolome results. (**a**) PCA plot. (**b**) PLSDA score plot. (**c**) PLSDA model validation plot. (**d**) Volcano plot of DAMs. (**e**) Clustering heatmap of DAMs. (**f**) KEGG enrichment scatter plot.

In the PCA plot (Fig. 3a), the cumulative values of the horizontal (PCA1) and vertical (PCA2) coordinates were 97.91% indicating that DAMs were present in the AP and Normal groups. In addition, the Partial Least Squares Discrimination Analyses (PLSDA) score plot explained 64.55% of the variation between the AP and Normal groups (Fig. 3b). The R2 value of the PLSDA model was 0.6858 and the Q2 value was less than 0, indicating that the PLSDA model was not overfit (Fig. 3c). Five DAMs (Supplementary Table S2) were observed between the AP and Normal groups (Fig. 3d). Furthermore, a clustering heatmap was drawn based on the expression levels of the DAMs (Fig. 3e). To further identify the roles of specific signalling pathways in AP behaviour, KEGG pathway analyses was performed on DAMs from AP and Normal ducks. A total of 13 KEGG pathways were significantly enriched in DAMs (Fig. 3f), including morphine addiction, cGMP-PKG signaling pathway, alcoholism, regulation of lipolysis in adipocytes, sphingolipid signaling pathway, vascular smooth muscle contraction, renin secretion, cAMP signaling pathway, parkinson disease, neuroactive ligand-receptor interaction, nucleotide metabolism, purine metabolism, and ABC transporters.

### Combined analysis of metabolome and transcriptome

**Fig. 4:**
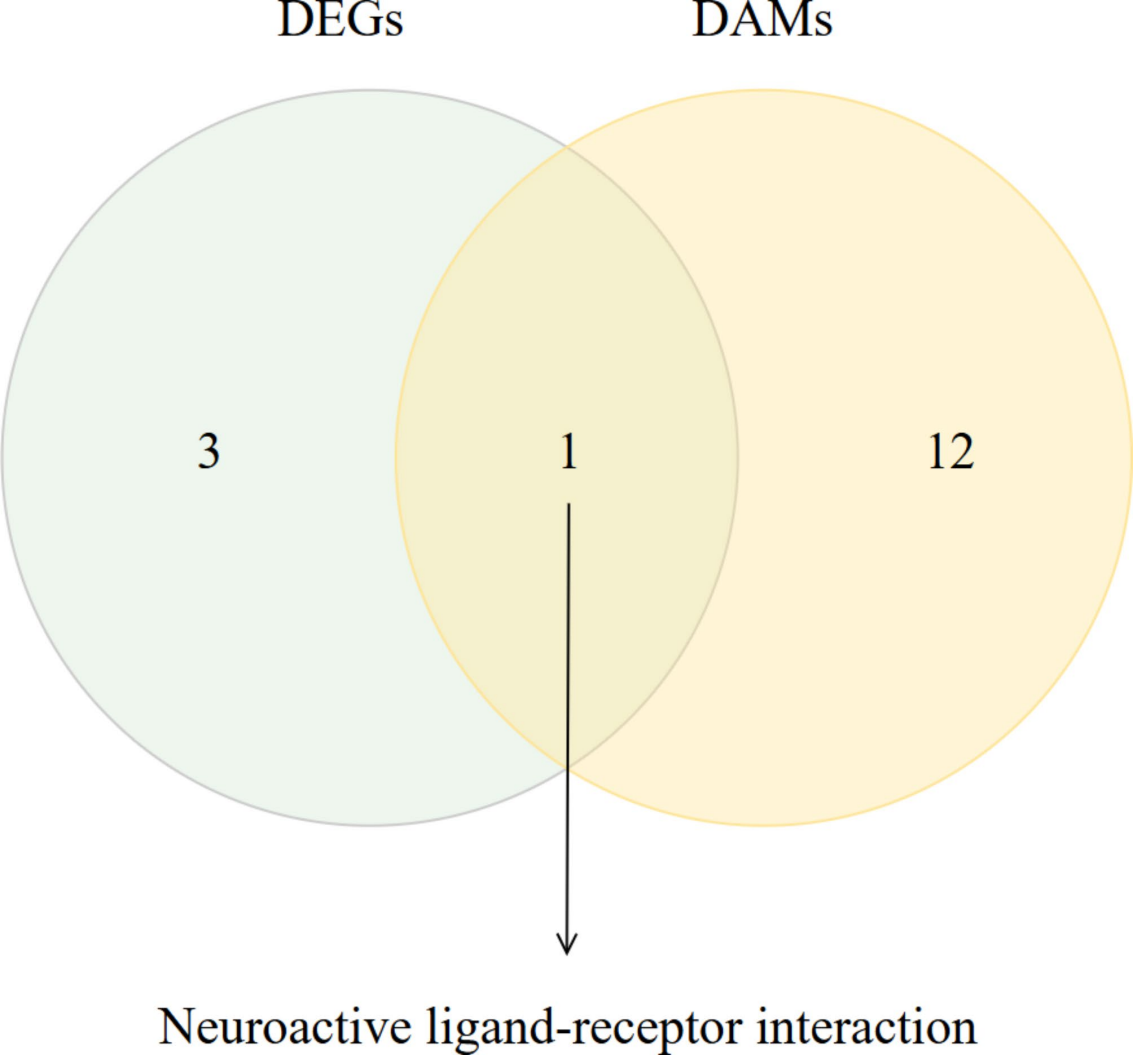
Venn diagram of KEGG significantly enriched pathways.

To further identify candidate genes and metabolites, the results of the transcriptome and metabolome were analyzed jointly. As shown in Fig. 4, neuroactive ligand-receptor interaction pathway was identical between DEGs and DAMs. Through the neuroactive ligand-receptor interaction pathway, 8 candidate genes including *ADCYAP1* (Adenylate cyclase-activating polypeptide 1), *GAL* (Galanin), *EDN2* (Endothelin 2), *EDN1* (Endothelin 1), *MC5R* (Melanocortin receptor 5), *S1PR4* (Sphingosine-1-Phosphate Receptor 4), *LOC113843450*, and *IAPP* (Islet Amyloid Polypeptide), and one candidate metabolite (adenosine) were identified (Table 2).

**Table 2:** KEGG pathway common to DEGs and DAMs.

Pathway	DEGs	DAMs
Neuroactive ligand-receptor interaction	*ADCYAP1*, *GAL*, *EDN2*, *MC5R*, *S1PR4*, *EDN1*, *LOC113843450*, *IAPP*	Adenosine

### qRT-PCR validation of RNA-Seq results

**Fig. 5:**
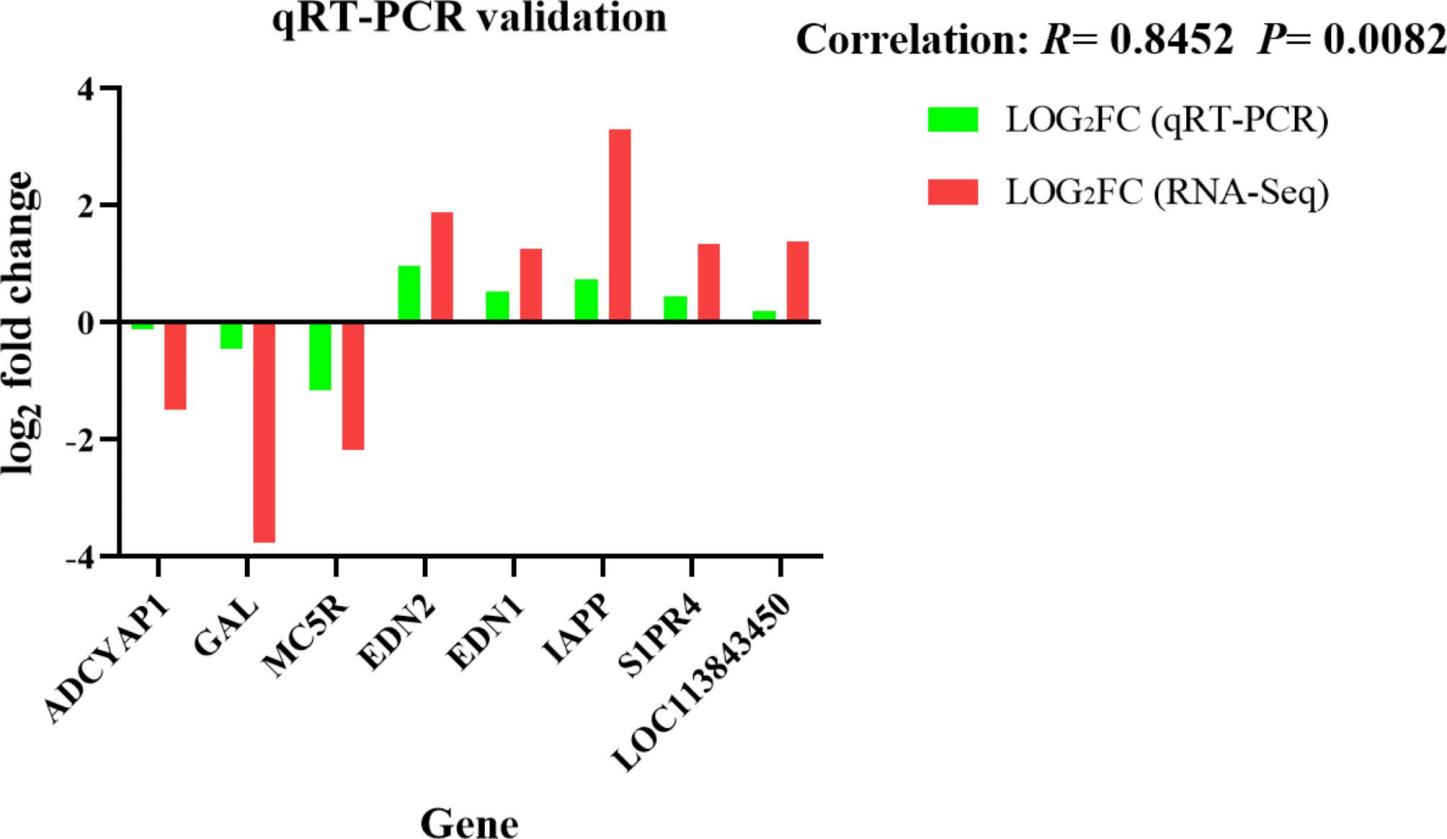
qRT-PCR validation.

To test the results of RNA-seq, 8 DEGs in the neuroactive ligand-receptor interaction pathway were analysed by quantitative real-time PCR (qRT-PCR) as shown in Fig. 5. The results showed that the expression trends determined by qRT-PCR were consistent with the RNA-Seq results, while a high correlation coefficient (*R* = 0.8452) and a significant *P* value (*P* = 0.0082) indicated that the RNA-seq results were reliable.

## Discussion

AP in poultry is an important way to establish and maintain social status. However, aggression also represents a source of stress for both dominant and subordinate animals^14^. Nerve cells are extremely sensitive to stress, and many studies have shown that stress causes progressive losses in nerve cell structure and function, affecting central nervous system (CNS) function and ultimately leading to many neurodegenerative disorders such as depression, Alzheimer’s disease, and dementia^15^. Patients with neurodegenerative disorders often exhibit cognitive deficits, hyperactivity, and high aggression^16^. In studies of birds, it has also been reported that stress causes damage to nerve cells, ultimately leading to a decrease in synaptic connections which affects an individual’s ability to learn, remember, and cope with stress^17,18^.

Nervous system dysfunction is considered an important factor that influences aggressive behaviour. A multitude of studies have reported that serotonin and dopamine, two key neurotransmitters, are involved in regulating aggressive behaviour^19^. However, no significant differences in the receptor genes of serotonin and dopamine were observed between the AP and Normal groups in our transcriptome results, which is in agreement with the results of a previous study in chickens^13^. Due to behavioural traits that are typically influenced by a large number of genes working together, we suppose that other alleles to some extent supersede the role of these neurotransmitter receptor genes in the regulation of AP. Additionally, Buitenhuis et al.^13^ demonstrated that GO terms such as synaptosomes and glutamate receptor binding may be related to AP behaviour by affecting memory formation. According to our transcriptome results, the most significantly enriched GO terms were translation, ribosomes, and structural components of ribosomes. Memory is a fundamental cognitive process that requires protein synthesis to consolidate memories^20^. Ribosomes are important macromolecular machines because they are associated with protein synthesis in cells. Previous studies have shown that neuronal protein synthesis occurs near synapses^21^. As a result of altered ribosome function, brain and behaviour changes, such as changes in memory functions, may follow in AP and Normal ducks. In addition, Lutz et al.^12^ showed that *GNG2* (G protein subunit gamma 2) may be related to the monomanine signaling pathway, which is involved in AP behaviour. Although *GNG2* was screened in our study, the log_2_(fold change) was 0.57 with a *p*-value of 0.15, which was not statistically significant. A comparison of our study results with literature reports revealed some inconsistent results, possibly because the test animals in the previous study were laying hens from a feather pecking selection line, whereas the test animals employed in our study were commercial ducks.

Furthermore, the combination of transcriptomic and metabolomic KEGG enrichment results in this study revealed one pathway, neuroactive ligand-receptor interaction, which was common to both the DEGs and the DAMs (Fig. 4). Previous studies have shown that neuroactive ligand-receptor interaction^22^ could influence memory formation. Through the neuroactive ligand-receptor interaction pathway, 8 candidate genes and 1 candidate metabolite were identified (Table 2). Neurodegeneration has been linked to impairments in memory and cognitive function^23^. Neuroinflammation is one of the hallmarks of neurodegeneration. Activated microglia and astrocytes produce proinflammatory factors and stimulate other immune cells to produce neurotrophic factors and inflammatory mediators^24^. However, overactivated microglia gradually shift from providing nutritional support and repairing nerve cells to becoming dysfunctional^25^. This response further recruits peripheral innate immune cells and adaptive immune cells across the blood-brain barrier, ultimately leading to cognitive and mood disorders^26^. *S1PR4* is a member of the sphingosine-1-phosphate receptor family, that is located in the active zone of nerve endings and regulates neuronal activity^27^. Furthermore, *S1PR4* can influence CNS function by modulating blood-brain barrier permeability^28^. *ADCYAP1* is a neuropeptide involved in neurotransmission in the CNS, and has also been shown to inhibit the production of proinflammatory factors by lipopolysaccharide-activated microglia to modulate the occurrence of neuroinflammation^29^. In addition, *ADCYAP1* can alleviate pain in the acute phase of peripheral nerve injury and promote the regeneration of nerve axons^30^. Multiple sclerosis is a chronic inflammatory disease of the CNS. *IAPP* is an amyloid polypeptide that can exert toxic effects by inducing neuroinflammation in the CNS^31^; *IAPP* can also lead to neuronal apoptosis by inducing mitochondrial dysfunction through the production of reactive oxygen species^32^. *GAL* is a class of neuropeptides that exert protective effects on the CNS. For example, under kainate (a neurotoxic amino acid) induced injury, the hippocampal neuronal cell death rate was greater in *GAL*-knockout mice than in wild-type mice, whereas in *GAL*-overexpressing mice, the hippocampal neuronal cell death rate was significantly lower than that in wild-type mice^33^. In addition, it has been shown that *GAL* knockout mice suffer from the loss of hippocampus-mediated spatial memory capacity^34^. The melanocortin system has been linked to memory, nociception, mood disorders and addiction^35^. *MC5R* is one of the receptors for melanocortin, and a study showed that *MC5R* expression was upregulated 3-4-fold in the hippocampal and midbrain regions of young rats compared with older rats^36^. This suggested cognitive decline occurs in rats with downregulated levels of *MC5R* expression. *EDN1* and *EDN2* are genes that encode endothelin and are associated with neurotransmission^37^. One study indicated that exposure of astrocytes to EDN1 and EDN2 significantly reduced the expression of glutamate transporter proteins^38^, whereas decreased glutamate transporter protein expression may increase glutamate levels^39^. Glutamate is an excitatory neurotransmitter in the CNS. High levels of glutamate can overstimulate neurons, leading to neuronal death^40^. In addition, *LOC113843450* has been reported in a few studies, though its effects on aggressive behaviour remain unclear.

Adenosine, a metabolite of adenosine triphosphate, is involved in the regulation of many physiological activities in the body. In the context of neurological injury, adenosine may exert neuroprotective effects; for example, the activation of the adenosine A_1_ receptor in ischaemie stroke mice enhances mitochondrial biogenesis and exerts neuroprotective effects^41^. Alternatively, adenosine can act as an inhibitory neuromodulator responsible for feedback regulation of excitatory synaptic activity. For example, the activation of adenosine A_1_ receptor at the presynaptic membrane inhibits the release of neurotransmitters such as glutamate^42^, 5-HT^43^, and dopamine^44^. In contrast, activation of the adenosine A_2a_ receptor promotes the release of glutamate, norepinephrine^45^ and 5-HT^46^.

In conclusion, the 8 candidate genes and adenosine enriched in the neuroactive ligand-receptor interaction pathway in the present study may affect the CNS function of AP ducks and Normal ducks by inducing neurodegeneration and disrupting the balance of neural excitation and inhibition, which might cause differences in the AP phenotype.

## Methods

### Animals, living conditions and AP observations

Thirty-six 17-week-old Sansui ducks were randomly divided into three cages (0.65 m H x 0.8 m W x 1.2 m D, 0.08 m^2^ per duck). Each duck was given a polyethylene collar of a different colour, while a camera was placed above the cage to record the number of AP. In this study, the observation of AP was divided into two phases: a preobservation period and a formal observation period, with a 14-day acclimatization period for each observation. The preobservation period was 3 days, and the number of AP was counted from 9:00 a.m. to 10:00 a.m. and from 15:00 p.m. to 16:00 p.m. During the preobservation period, a duck was defined as an AP duck when it gave more than one AP, and a duck was defined as a Normal duck when it gave zero AP. After preobservation, 12 AP ducks and 12 Normal ducks were then divided into two cages for formal observation, and the number of AP was counted by video for seven consecutive days (every day from 9:00 a.m. to 10:00 a.m. and from 15:00 p.m. to 16:00 p.m.). At the end of the formal observation period, three AP ducks with the highest number of occurrences of AP were selected, and three Normal ducks without AP were selected. The animals were subsequently euthanized by cervical dislocation, and their whole brains were homogenized separately using a tissue homogenizer (QK-1B, Jingxin, China), and preserved at -80℃ for transcriptomic and metabolomic analyses. In this study, the ducks were fed twice a day (8 a.m. and 17 p.m.), had free access to water, and were maintained under a 18 L: 6D photoperiod.

### RNA extraction, cDNA library construction, and sequencing

The total RNA of 6 whole brain samples, 3 from Normal group and 3 from AP group, were extracted with TRIzol reagent (15596018, Thermo Fisher, USA). The total RNA quantity and purity were analyses with a Bioanalyzer 2100 (Agilent, CA, USA) and a NanoDrop ND-2000 (NanoDrop, Wilmington, DE, USA), and RNA samples with RNA integrity numbers > 7.0 were used to construct sequencing library. After total RNA was extracted, mRNA was purified from the total RNA using Dynabeads Oligo (dT) _25_ (61005, Thermo Fisher, CA, USA) through two rounds of purification. After purification, the mRNA was fragmented into short fragments using a Magnesium RNA Fragmentation Module (E6150S, NEB, USA). Then the short RNA fragments were reverse-transcribed to cDNA with SuperScript™ II Reverse Transcriptase (1896649, Invitrogen, USA), and the cDNA was used to synthesise U-tagged second-strand DNAs with DNA polymerase I (M0209, NEB, USA), RNase H (M0297, NEB, USA) and dUTP Solution (R0133, Thermo Fisher, USA). An A base was added to the blunt end of each strand in preparation for ligation to the index adapters, as each adapter contains a T base pendant for ligating the adaptor to the A-tailed fragment DNA. Dual-index adapters were ligated to the fragments, and size selection was performed with AMPureXP beads. After heat-labile UDG enzyme (M0280, NEB, USA) treatment of the U-labelled second-stranded DNAs, the ligated products were amplified by PCR. The average insert size for the cDNA library was 300 ± 50 bp. Finally, paired-end sequencing was performed on an Illumina Novaseq™ 6000 (Illumina, USA) according to standard operation in PE150 sequencing mode. RNA sequencing was carried out by LC Bio Technology Co.,Ltd.

### RNA-seq data analyses

Further filtering of the reads was performed using Cutadapt (V1.9) and sequence quality was verified in FastQC (V0.11.9). Afterwards, we aligned reads from all samples to the *Anas platyrhynchos* reference genome using HISAT2 (V2.2.1) and transcriptome was assembled for each individual sample using StringTie (V2.1.6), followed by a composite transcriptome reconstruction using gffcompare (V0.9.8). Transcript expression levels and FPKM values were determined using StringTie and Ballgown (V2.30.0), and differential expression analyses were performed based on the FPKM values using DESeq2 software (V1.10), with thresholds of *P-*values < 0.05 and |log_2_(fold change)| ≥ 1 to identify genes as DEGs. The DEGs were subsequently subjected to enrichment analyses of GO functions (http://www.geneontology.org/) and KEGG pathways^47^. PCA was performed with the princomp function of R software (V 3.6).

### Metabolite extraction and nontargeted metabolomics

Fifty milligrams of each whole brain sample, 3 from Normal group and 3 from AP group, was separately added to a centrifuge tube with 500 µL of precooled 80% methanol (A-456-4, Thermo Fisher, USA) and homogenized (50 Hz) for 60 s. The centrifuge tube subsequently was placed at -20 ℃ for 30 min, and then centrifuged at 4 ℃ and 20,000 g for 15 min. The supernatant was transferred to another centrifugal tube and vacuum dried. Then, the supernatant was redissolved in 100µL of 80% methanol, and centrifuged at 4 ℃ and 20,000 g for 15 min and stored at -80 °C prior to liquid chromatography-mass spectrometric (LC-MS) analyses. In addition, pooled QC samples were also prepared by combining 10 µL of each extraction mixture. LC-MS was carried out by LC Bio Technology Co.,Ltd.

LC analyses was performed on a Vanquish Flex UPLC System (Thermo Fisher, Germany) and chromatography was carried out with an ACQUITY UPLC T3 (100 mm × 2.1 mm, 1.8 μm, Waters, UK). The column temperature was 40 °C, and the flow rate was 0.30 mL/min. Mobile phase A was 5 mmol/L ammonium acetate plus 5 mmol/L acetic acid, and mobile phase B was acetonitrile. The gradient elution conditions were set as follows: 0–0.8 min, 2% B; 0.8–2.8 min, 2–70% B; 2.8–5.3 min, 70–90% B; 5.3–5.9 min, 90–100% B; 5.9–7.5 min, 100% B; 7.5–7.6 min, 100–2% B; and 7.6–10.0 min, 2%. MS detection of metabolites was performed on a Q Exactive instrument (Thermo Fisher, Germany), which was operated in positive (4000 V) and negative (4500 V) ionization modes, and the temperature of the electrospray ionization source was 350 °C. Precursor spectra were acquired at a resolution of 70,000 to achieve an AGC target of 3e6. The maximum injection time was set to 100 ms. The top 3 signal ions with signal accumulation intensities above 100,000 were then selected from the primary spectrum for a secondary fragmentation scan. The resolution of the secondary level was 17,500 and the maximum injection time was set to 50 ms.

### Metabolome data analyses

The MS data were preprocessed in the XCMS package of R software for peak picking, peak grouping, retention time correction, and second peak grouping. The metabolites were characterized by combining the retention time and mass/ charge number of ion data for each ion using the KEGG database. The criterion for significant DAMs selection was *P* value < 0.05.

PCA was performed using metaX package of R software, PLSDA was performed in the ropls package, and hierarchical clustering was performed using the pheatmap package.

### Validation of RNA-seq Data by qRT-PCR

To test the reliability of the RNA-seq results, 8 DEGs in the neuroactive ligand-receptor interaction pathway were selected for qRT-PCR analyses. *GAPDH* (Glyceraldehyde-3-phosphate dehydrogenase) was used as an internal marker to normalize the expression levels. The primers used for qRT-PCR are listed in Table 3. Total RNA samples were reverse transcribed to cDNA using a RevertAid™ First Strand cDNA Synhesis Kit (Thermo Fisher, K16225). Gene expression was analyzed using a CFX96 (Bio-Rad, USA) and 2×RealStar Fast SYBR qPCR Mix (Genstar, A301). Each sample was analyzed in triplicate and relative quantification of gene expression was performed using the 2^−ΔΔCt^ method.

**Table 3:** Primers for qRT-PCR.

Gene	Reference number	Primers (5′-3′)
*ADCYAP1*	XM_027451981.2	F: TAATAATGCATTGCAGCGTC R: GGCGTCCTTTGTTTTTAACT
*GAL*	XM_027459416.2	F: CTTGGGCCACGTCGTAT R: TCAAGACTGGTTTGTTTCCTC
*IAPP*	XM_005023030.5	F: TCGTGTCACAGAATACACTC R: TGTGCATTGTTTTGGAGTTC
*EDN2*	XM_027443949.2	F: TATCTGGGTCAACACACCT R: GCATTCCTCTGAGAATAGCG
*MC5R*	XM_038175186.1	F: TCTGAACTAAACCTGAGTGC R: ACAGTGAGACCATGAGAAAC
*EDN1*	XM_027451476.2	F: AGACCGTTCCTTATGGTCTT R: TTTAAGCTTCCACCCTTTCT
*LOC113843450*	XM_038178002.1	F: CTACATCCAGAACTGCCC R: AGCACCGTCAGGTTCTT
*S1PR4*	XM_005019523.5	F: TCTAATCTCTGCCTCTCAGG R: CAATAATGAAGGCCAGAAGC
*GAPDH*	XM_038180584.1	F: GGTTGTCTCCTGCGACTTCA R: TCCTTGGATGCCATGTGGAC

### Statistical analyses

The qRT-PCR data were statistically analysed and plotted using Excel 2021 and GraphPad Prism (V8.0). The Pearson correlation coefficient was used to analyze the correlation between RNA-seq and qRT-PCR data. The Data were expressed as mean ± SDs. Student’s t-test was used to analyze the differences in the RNA-seq data and metabolome data. *P* < 0.05 indicated statistical significance.

## Electronic supplementary material

Supplementary Table S1.

Supplementary Table S2.

## Data Availability

The sequence reads are available from the National Center for Biotechnology Information (NCBI) Bioproject database (Ascension number PRJNA1122767). The other datasets used and analyzed during the current study are available from the corresponding author on reasonable request.
